# Origin, distribution, and perspective health benefits of particulate matter in the air of underground salt mine: a case study from Bochnia, Poland

**DOI:** 10.1007/s10653-021-00832-2

**Published:** 2021-02-11

**Authors:** Aleksandra Puławska, Maciej Manecki, Michał Flasza, Katarzyna Styszko

**Affiliations:** 1 Department of Mineralogy, Petrography and Geochemistry, Faculty of Geology, Geophysics and Environmental Protection, AGH University of Science and Technology, Al. Mickiewicza 30, 30-059 Kraków, Poland; 2 Bochnia Salt Mine, ul. Campi 15, 32-700 Bochnia, Poland; 3 KGHM CUPRUM Ltd. R&D Centre, ul. Sikorskiego 2-8, 53-659 Wrocław, Poland; 4 Department of Coal Chemistry and Environmental Sciences, Faculty of Energy and Fuels, AGH University of Science and Technology, Al. Mickiewicza 30, 30-059 Kraków, Poland

**Keywords:** Halotherapy, Saline aerosol, Ambient and indoor air pollution, Salt mine, Subterranean airborne particles, Speleotherapy

## Abstract

The composition and distribution of airborne particles in different locations in a salt mine were determined in terms of their origin, the distance from the air inlet, and the adaptation of post-mining chambers and corridors for tourists and general audience. The composition of aerosols in air was also evaluated from the perspective of human health. Air samples were collected on filters by using portable air pumps, in a historical underground salt mine in Bochnia (Poland), which is currently a touristic and recreation attraction and sanatorium. The particulate matter (PM) concentration was determined using the gravimetric method by weighing quartz filters. The content of carbon, water-soluble constituents, trace elements, and minerals was also determined. A genetic classification of the suspended matter was proposed and comprised three groups: geogenic (fragments of rock salt and associated minerals from the deposit), anthropogenic (carbon-bearing particles from tourist traffic and small amounts of fly ash, soot, and rust), and biogenic particles (occasional pollen). The total PM concentration in air varied between 21 and 79 μg/m^3^ (with PM_4_ constituting 4–24 μg/m^3^). The amount of atmospheric dust components coming from the surface was low and decreased with the distance from the intake shaft, thus indicating the self-cleaning process. NaCl dominated the water-soluble constituents, while Fe, Al, Ag, Mn, and Zn dominated the trace elements, with the concentration of majority of them below 30 ng/m^3^. These metals are released into air from both natural sources and the wear or/and corrosion of mining and tourists facilities in the underground functional space. No potentially toxic elements or constituents were detected. The presence of salt particles and salty spray in the atmosphere of salt mine, which may have anti-inflammatory and antiallergic properties, is beneficial to human health. This study will allow for a broader look at the potential of halotherapy in underground salt mines from a medical and regulatory point of view.

## Introduction

Particulate matter (PM) suspended in the atmosphere is a complex mixture of solid and liquid particles. Typical PM consists of inorganic compounds, organic carbon (OC), elemental carbon (EC), mineral dust, and biological materials and is often accompanied with toxic metals and compounds (Ghasemi et al. 2020). It can be of natural origin (e.g., sea spray, aeolian products of soil and crust erosion, volcanic emissions, vegetation, and microorganisms) and can also be a product of anthropogenic processes (e.g., combustion of fossil fuels, industrial emissions, transportation emissions, and emissions from construction projects) (Goudarzi et al. 2019; Jabłońska and Janeczek 2019). In the indoor environment, PM concentrations are influenced by other sources such as emissions from cooking, smoking, heating, human presence and activity, and use of room equipment and household appliances (Rivas et al. 2019). In addition, PM occurring within enclosed spaces includes particles of external origin that migrate indoors (Grau-Bové and Strlič 2013; Cong et al. 2018). In many cases, the concentration of PM in indoor is higher than that in outdoor spaces (McCormack et al. 2008; Kim et al. 2015; Mainka et al. 2015). The potential impact of PM on human health can vary widely according to their size and the mineral and chemical composition of the inhaled particles. In general, the finer a particle, the deeper it penetrates the respiratory system and the fraction < 4 µm is commonly accepted as a respirable fraction (Trechera et al. 2020; Kim et al. 2015).

The composition of particles in the air of underground mines depends primarily on the type of mineral, type and intensity of the mining processes, and ventilation systems (Saarikoski et al. 2018). For decommissioned, open-access mines, the factors that influence the composition of the atmosphere depend less on mining activities and more on their use for museum, tourism, recreation, and health purposes. Weathering of parent rocks in a mine is a source of aerosols and dusts that supply natural geogenic components to the underground atmosphere. On the other hand, the presence of visitors and tourist services introduce particles typical for air indoors (Alves et al. 2014; Gębarowska et al. 2018). Because underground air is actually the ambient air that is drawn in from the surface, the composition of PM in the mine is also dependent to some extent on particles from the ambient air (Salmon et al. 1996). In addition, the mine’s complex ventilation system creates specific conditions that are difficult to compare with other environments. It is therefore very important to define tools, techniques, and methods for testing airborne particulates in relation to specifics of public utility environments (Śmiełowska et al. 2017).

Faced with the challenge of increasing urban air pollution and increased drug resistance, complementary methods for the treatment of chronic respiratory infections and advanced post-treatment convalescence methods such as halotherapy are being intensively investigated (Hedman et al. 2006; Sandu et al. 2011, 2015; Rashleigh et al. 2014; Bar-Yoseph et al. 2016). Halotherapy is derived from speleotherapy, which is a drug-free respiratory therapy that involves breathing inside a cave. This method is based on the effect of natural therapeutic factors occurring in a specific environment on the human body (Horvath 1986; Liu et al. 2018). The beneficial effect of salt spray was noticed as early as 1843, when one of the Polish physicians noted that employees of salt mines do not have respiratory problems or suffer from lung diseases (Chervinskaya and Ziber 1995). Since then, underground salt spray treatment has been constantly developing in many Central and Eastern European countries such as Poland, Austria, Germany, Romania, Russia, Ukraine, and Belarus (Horowitz 2010; Simionca 2013; Ernst 2019).

Because of the beneficial microclimate, historical significance, and relatively stable geological and mining conditions, decommissioned salt mines are the favored location to open sanatoriums, touristic hiking trails, and museums. Moreover, the development of various types of mining tourism, including medical tourism, has a high social and economic value in the process of re-profiling post-mining areas (Kaźmierczak et al. 2019). The popularity of such facilities is apparent even in modern salt mining, which often combines traditional rock salt mining with the opening of parts of the mine to the public: see, for example, Kłodawa (Poland), Praid (Romania), Soligorsk (Belarus), and Loulé (Portugal). Simultaneously, a rapid expansion of artificial analogies of speleotherapy (halochambers) is observed in many places worldwide (Chervinskaya 2003; Lazarescu et al. 2014; Zając et al. 2014; Achkar et al. 2015).

The air in salt mines is characterized by high microclimate quality, high concentration of sodium chloride, high microbiological purity, negative ionization, and low level of natural radioactivity (Calin et al. 2012; Kostrzon et al. 2015a; Wiszniewski 2015). Therapeutic stay in salt mines is recommended for people suffering from chronic disorders such as bronchial asthma and chronic obstructive pulmonary disease (Obtułowicz 2013; Kostrzon et al., 2015b, 2019; Mętel et al. 2020). Although the mechanism responsible for the therapeutic effects of subterranean therapy on the human body has not yet been explained in detail, the presence of salt spray in the atmosphere of sanatoriums in salt mines is definitely considered to be beneficial.

Although some authors consider halotherapy to be an uncertain and unproven treatment (Shah and Greenberger 2012; Sandell et al. 2013), sanatorium and halotherapy activities continue to be developed in salt mines (Simionca 2013; Kostrzon et al. 2019; Mętel et al. 2020). Recent studies on the discussion of the suitability of the underground aerosol of salt mines in respiratory treatment have focused mostly on bioaerosol and microbiological purity in the atmosphere of salt mines (Frączek and Grzyb 2010; Grzyb and Frączek 2010; Frączek et al. 2013; Gębarowska et al. 2018; Myszkowska et al. 2019). Only few studies have investigated the composition of inorganic solids (Salmon et al. 1996; Rogula-Kozłowska et al. 2016). This is surprising given the fact that inorganic aerosol is considered to be a therapeutic factor in halotherapy. Recently, Rogula-Kozłowska et al. (2016) reported on the concentration and chemical composition of PM collected at selected points of the underground Wieliczka Salt Mine. However, published information on the diversity of mineral components present in the air, especially those from the surrounding rocks, is still scarce, and there is a lack of such systematic research in underground salt mines. The results of such research will allow to indicate the most significant differences between salt spray on the seashore, artificial halochambers on the surface, and saline aerosol in the atmosphere of an underground salt mine. The present study broadens the knowledge of the composition and distribution of airborne particles in different locations in a salt mine in terms of their origin, the distance from the air inlet, and the use of post-mining chambers and corridors for tourists and general audience. On the basis of a carefully designed sampling strategy, the characteristics of mineral and chemical diversity of airborne particles and their spatial distribution in a salt mine were determined. The effect of particles present in the atmospheric air sucked in from the surface to the ventilation system was assessed, along with the role of tourist traffic and regular mine service. The composition of mine aerosols was also evaluated from the perspective of human health. The authors believe that this study will allow for a broader look at the potential of halotherapy in underground salt mines.

## Materials and methods

### Site characteristics

The composition and distribution of airborne particles in various locations in a salt mine were determined in terms of their origin, the ventilation system, the distance from the air inlet, and the use of underground corridors. The research was conducted in the Bochnia Salt Mine, located near Kraków in southern Poland. The beginning of rock salt mining in Bochnia dates back to the first half of the thirteenth century. The production of salt ended in 1990. Since then, similar to other historical salt mines in the world, the Bochnia Salt Mine has focused its activities on tourism, recreation, and health care. Because of its cultural and natural importance to the common heritage of mankind, it is enlisted in the UNESCO World Heritage List.

**Fig. 1:**
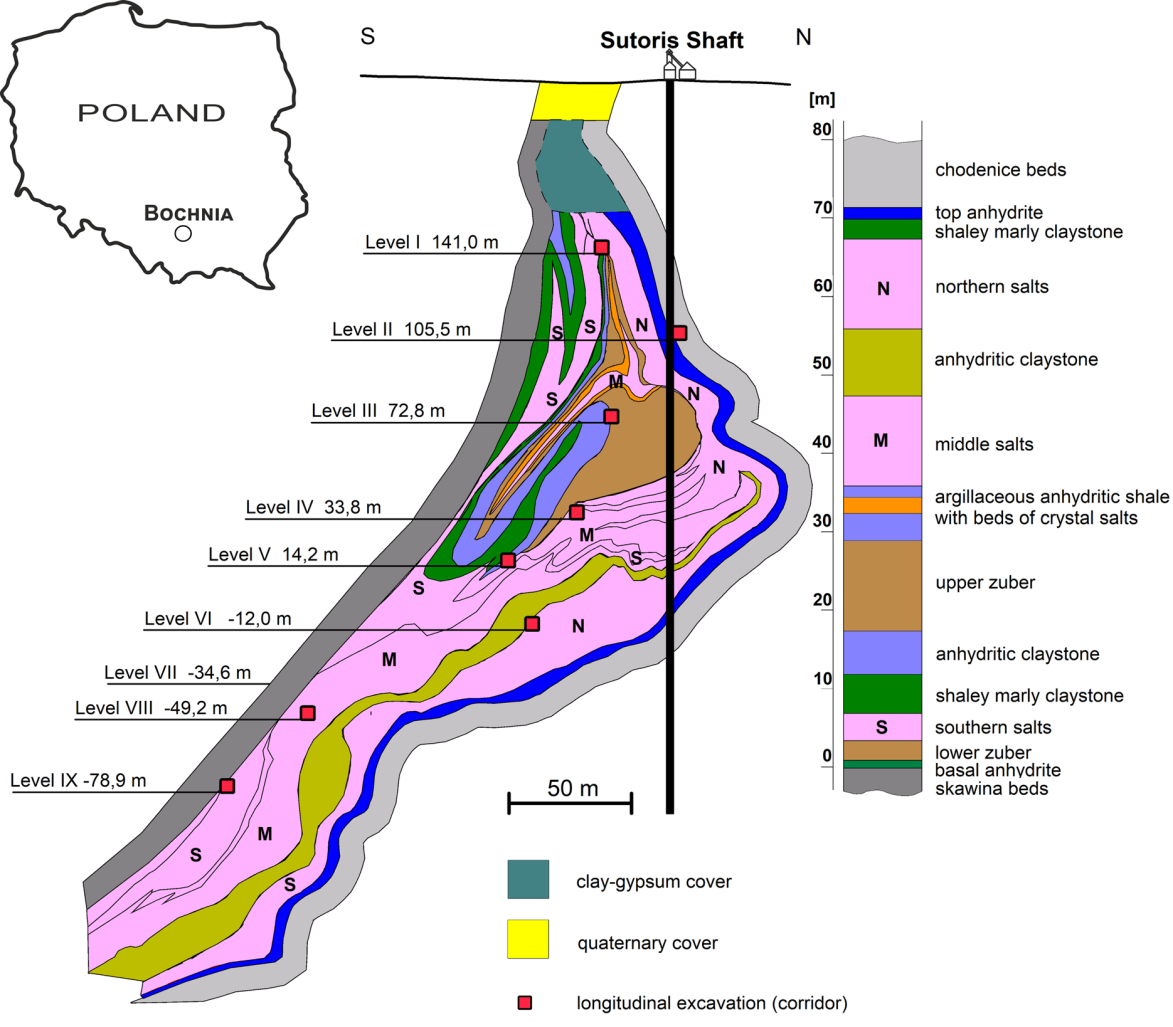
Geology of Bochnia salt deposit: a simplified structure of a cross section around the Sutoris shaft (Poborski 1952; Wagner 2010; modified) and a lithostratigraphic profile of salt succession (according to Poborski 1952, modified). The depth of corridors is given in meters above sea level

The Bochnia Salt Mine was established in a fragment of the marine sediments of the Miocene salt-bearing formation. The Bochnia salt deposit was formed due to tectonic enrichment of initially thin rock layers of salt. There are three units of salt layers: southern, middle, and northern (Fig. 1). The southern and middle salts are mainly gray, medium, and coarse-grained. Middle salts are sporadically represented by very thin layers of salt with interlayers of clayey anhydrite. The northern salts consist of alternating layers of fine-grained salt and claystone with anhydrite. Rock salt occurs in layers with a maximum thickness of 1 m, is evenly contaminated with clay and anhydrite, and locally contains layers of pure salt (Wagner et al. 2010). Geological setting of this salt deposit is relatively well characterized (Poborski 1952; Garlicki 2008; De Leeuw et al. 2010).

As a result of many years of mining activity, the layers of pure southern and middle rock salt have been almost completely extracted. Halite with an intercalation of clay, anhydrite, and gypsum is observed on most of the mine sidewalls and ceilings. Mining supports are used in excavations where gangue rocks (clay, anhydrite) comprise a substantial area of the excavation site. Timber supports are mainly used, while steel arch supports are sporadically used.

**Fig. 2:**
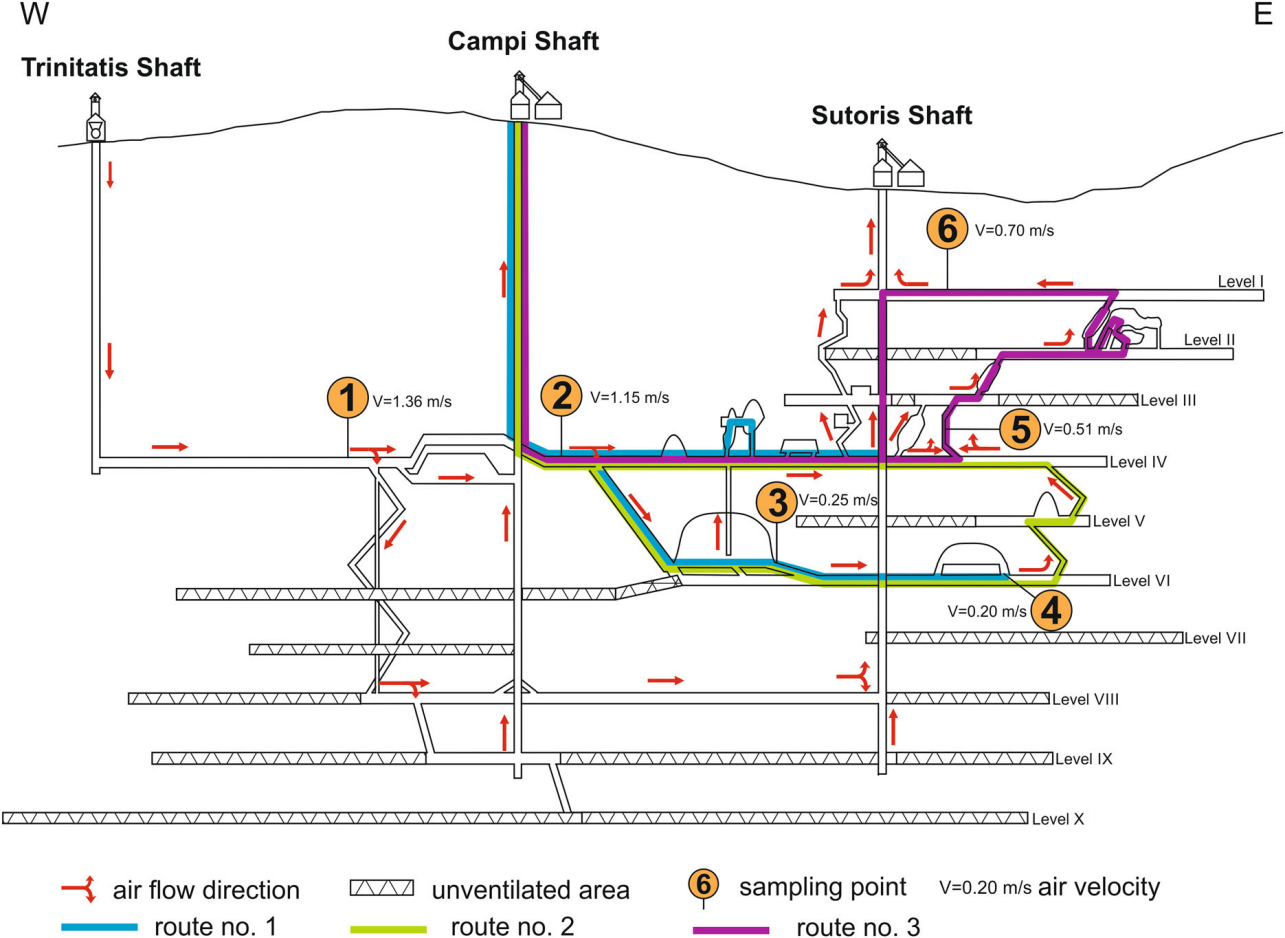
Location of the sampling sites in relation to tourist traffic and air flow directions on a schematic longitudinal section of Bochnia Salt Mine

The current extensive spatial structure of the historic mine consists of 9 post-mining galleries extending down to 350 m below the surface. A significant part of the excavations is open to the public (Fig. 2). The number of visitors to the Bochnia Salt Mine reaches up to 180,000 per year.

The mine is ventilated by a system of three connected shafts: the nineteenth-century Trinitatis (inspiratory), the sixteenth-century Campi (exhaust), and the thirteenth-century Sutoris (exhaust). Two fans working continuously on the surface force the air into the mine. On average, 1500 m^3^ of air per minute is transferred through 210 long shaft Trinitatis (Fig. 2). The air is distributed through the mine through internal channels, and its flow is controlled by regulators and fans so that the concentration of oxygen in the air in all workings in a tourist zone is not less than 20.85%. The efficiency of the mining ventilation is constantly controlled by a remote measurement and alarm system supervised by mine personnel and confirmed by laboratory analyses as required by law.

### Tourist traffic in the mine

Tourists go down to the mine and leave the mine through the Campi shaft. The tours take place in groups supervised by a guide. Three different routes are provided for the visitors (Fig. 2). All groups are transported from the Campi shaft to the beginning of a selected route by underground train. The main and most crowded route is route no. 1 leading along excavations of level IV and VI. The last stage of this tour is the stay in Ważyn Chamber, lasting at least half an hour for each tourist group. Ważyn Chamber is the largest chamber in the mine (over 250 m long) and has been arranged as a multifunctional space with an underground kitchen, playground and a bedroom with up to 250 beds. The next route (no. 2) leads through level IV, V, and VI and also includes a stay in the Ważyn Chamber. Tourist route no. 3 leads through the oldest mining excavations near the Sutoris shaft, where salt was mined from the Middle Ages to the early twentieth century.

### Sampling protocol

Although the Bochnia Salt Mine is a closed mine, it is still regulated by the Polish Geological and Mining Law to ensure the safety of people underground, both personnel and visitors. Therefore, Gillian pumps (Sensidyne, USA), certified for use in Polish underground mines, were used to collect samples in this research. Inhalable dust sampler for TPM and cyclone sampler for PM4 provided by Sensidyne were applied. In order to determine the origin, distribution, and prospective health benefits of airborne PM in an underground salt mine, a number of airborne PMs were collected at six different locations in the mine. The sampling points were arranged along the main air stream in the order of increasing distance from the air intake of the mine. PM was collected along public mine passages and in front of the tourist zone. Points 2 to 6 are located on tourist trails. The highest tourist traffic is in points 2 and 3. The location of sampling sites is presented in Fig. 2.

Sampling point number 1 is located approximately 880 m from the Trinitatis shaft (inspiratory shaft) at level IV (212 m below the ground surface). This is a tunnel that supplies air to the rest of the mine. It is carved mostly in northern salt. Point number 2 is further away, 1480 m from the intake shaft, in the same tunnel at level IV. It is located in the corridor used by a train transporting tourists from the Campi shaft (the entrance) to the main tourist route. Samples at point number 3 were collected on a ramp at the end of the largest chamber in the mine—the Ważyn chamber. This chamber, situated at level VI (248 m below the ground surface), is made from northern and middle salts. The air travels a distance of 1890 m from the inspiratory shaft. Sampling site number 4 is located on level VI, near a 120-m long underground brine lake (distance from intake shaft equal to 2505 m). In the sample site number 5, two different air paths merge: from the western part of the mine (level IV, 2380 m from the inlet well) and from the eastern part (level VI, 3010 m from the inlet well). This air is then distributed to the upper part of the mine. Sampling site 6, the furthest point from the inspiratory shaft (distance from site 5 is approximately 1150 m), is on Level I located 70 m below the ground surface.

The instruments were installed in the middle of the ceiling with an inlet 1.6–1.8 m above the floor. This was necessary to ensure the smooth operation of the air samplers regardless of the presence of visitors in the mine. The research was conducted in June 2019. The measurements were carried out in accordance with the general standards defining the conditions for conducting measurements in workplaces (e.g., PN-91/Z-04030/051,991; PN-91/Z-04030/06 1991). The sampling was performed in 4 series (Table 1). A single measurement was set to a time interval of 48 to 72 h at an air flow rate of 2–2.2 dm^3^/min. Thus, 24 samples of total particulate matter (TPM) and 6 samples of respirable particulate matter (PM_4_ with aerodynamic diameter below 4 µm) were collected. Due to the possible negative impact of long sampling times on quartz filters, field blanks were applied. Simultaneously, air temperature and relative humidity (RH) were determined at each sampling point. Samples for TPM and PM_4_ were collected at each location for 48 and 72 h, respectively. Polycarbonate (PC) filters of 25 mm diameter and 0.8 µm pore size were used (Whatman Nuclepore). A series of 72-h TPM samples were then taken using quartz filters (Whatman QMA, ϕ25 mm). Additionally, settled airborne dust particles were collected at sampling point 1. The samples were brushed from flat surfaces into a sealed container and prepared for mineralogical analysis of solids.

**Table 1:** Description of the sampling protocol

No. of sampling series	Date of measurements	Time interval [h]	Filter type	PM fraction	Application
1	2019/06/7–10	72	QMA	TPM	gravimetry, OC/EC
2	2019/06/10–13	72	QMA	TPM	gravimetry, water-soluble components IC
3	2019/06/13–16	72	QMA	TPM	gravimetry, microelements ICP-MS
4	2019/06/16–18	48	PC	PM4	gravimetry, SEM/EDS
	2019/06/16–19	72	PC	TPM	gravimetry, SEM/EDS

### Analytical methods

The collection of airborne PM was performed in 4 sampling series (Table 1). The PM concentration was determined using the gravimetric method by filter weighing on a microscale (Radwag, Poland, resolution of 10 µg). The filters were conditioned at 45% ± 5% of RH and 20 ± 2 °C prior to and after sampling and weighed at least three times to obtain a constant value, and the average value was then determined.

The content of organic carbon (OC) and elemental carbon (EC) was determined by the thermal-optical method using the NIOSH protocol (Sunset Laboratory, OR, USA) from samples collected on quartz filters during the first sampling series.

Water-soluble cations (Na^+^, K^+^, Mg^2+^, Ca^2+^, and NH_4_^+^) and anions (Cl^−^, NO_2_^−^, NO_3_^−^, SO_4_^2−^ PO_4_^3−^, Br^−^ and F^−^) were extracted from the samples collected on quartz filters (the second sampling series, Table 1) and analyzed by ion chromatography (IC). The filters were divided into two halves and extracted by ultrasonic agitation for 20 min in 1.5 mL of extra pure water (to determine the concentrations of anions) or in 1.5 mL of 12 mM methanesulfonic acid (to determine the concentrations of cations). The solutions were analyzed using an ICS-1100 instrument (Thermo Scientific) equipped with an autosampler AS-DV.

A third sampling series (Table 1) was used to analyze microelements. The elements were extracted from QMA quartz filters using hot acid digestion with concentrated HNO_3_ and HCl solutions at 4:1 ratio. The extracts were analyzed using inductively coupled plasma-mass spectrometry ICP-MS (ELAN 6100; PerkinElmer). A total of 28 elements were analyzed: Al, Fe, Mn, Ti, Ag, As B, Ba, Be, Cd, Co, Cr, Cu, Hg, Li, Mo, Ni, Pb, Rb, Se, Sn, Sr, Tl, U, V, W, Zn, and Zr.

Morphological and chemical characterization of PM was performed with a scanning electron microscopy (SEM, FEI Quanta model 200 FEG) equipped with an energy-dispersive spectroscopy (EDS) microanalyzer. A representative fragment of 0.5 cm^2^ of each polycarbonate filter (TPM and PM_4_) was cut and fixed on the sample holder with a carbon tape. The samples were coated with gold prior to imaging.

Mineral composition of PM was determined from samples of settled dust by X-ray diffractometry (XRD). The patterns were recorded with a Rigaku SmartLab diffractometer (Neu-Isenburg, Tokyo, Japan) in the range of 2–75°2θ with a step size of 0.05° using graphite monochromatized Cu Kα radiation. The phases were identified using the ICCD database and XRAYAN software (Marciniak et al. 2006).

Air temperature and RH were determined by an Assmann psychrometer during the second sampling campaign. The dry bulb and wet bulb temperatures were recorded to calculate RH.

Field blank samples were prepared to determine the analyte background levels and potential contamination during handling, transport, and storage procedure. The limits of quantification (LOQ) for chemical analyses are presented in Table 2. All results reported in this paper were blank corrected.

**Table 2:** Concentration of carbonaceous fraction, water-soluble fraction, and microelements associated with PM in the air of the Bochnia Salt Mine at various sampling sites

	LOQ	1	2	3	4	5	6
	Carbonaceous and water-soluble fraction, µg/m^3^						
OC	0.02	0.87	2.37	7.42	2.73	1.84	2.41
EC	0.02	0.13	0.25	0.60	0.05	0.24	0.68
TC	–	1.01	2.11	8.08	2.81	2.09	3.09
Na^+^	0.04	0.79	1.43	2.43	0.48	1.69	3.17
NH_4_^+^	0.03	0.34	0.14	bdl	bdl	bdl	bdl
Mg^2+^	0.03	0.08	0.14	0.54	0.07	0.07	0.03
K^+^	0.04	0.06	0.08	0.15	0.05	0.33	0.67
Ca^2+^	0.09	0.34	0.18	1.55	0.15	0.21	0.09
F^−^	0.12	bdl	bdl	bdl	bdl	bdl	bdl
Cl^−^	0.59	1.65	1.33	3.72	0.60	1.81	6.42
NO_2_-	0.02	0.02	0.02	0.02	0.02	0.02	bdl
Br^−^	0.02	bdl	bdl	bdl	bdl	bdl	0.06
NO_3_-	0.02	0.10	0.05	0.02	bdl	bdl	bdl
PO_4_^3−^	0.14	2.41	2.16	2.79	4.21	3.57	3.33
SO_4_^2−^	0.06	3.39	3.71	3.77	0.93	1.74	1.60
	Microelements, ng/m^3^						
Al	28.9	231.15	142.21	1017.07	bdl	351.9	bdl
Fe	57.8	1053.06	10,209.22	5389.08	230.67	1348.93	bdl
Mn	17.3	23.02	98.97	65.66	bdl	18.32	bdl
Ti	57.8	bdl	bdl	96.67	bdl	bdl	bdl
Ag	5.8	2.38	264.72	57.08	152.12	113.35	177.28
As	5.8	bdl	bdl	bdl	bdl	bdl	bdl
B	28.9	35.92	bdl	37.22	bdl	bdl	bdl
Ba	2.9	bdl	bdl	49.29	bdl	10.30	bdl
Be	2.9	bdl	bdl	bdl	bdl	bdl	bdl
Cd	1.7	bdl	bdl	bdl	bdl	bdl	bdl
Co	1.2	bdl	bdl	0.10	2.12	1.14	1.87
Cr	5.8	bdl	bdl	bdl	bdl	3.85	12.47
Cu	5.8	bdl	1.02	bdl	12.08	13.66	21.10
Hg	0.6	bdl	bdl	bdl	bdl	bdl	bdl
Li	28.9	bdl	bdl	bdl	bdl	bdl	bdl
Mo	1.7	bdl	bdl	bdl	bdl	bdl	bdl
Ni	5.8	bdl	6.13	3.77	8.59	8.60	13.26
Pb	0.6	bdl	bdl	bdl	bdl	bdl	bdl
Rb	0.6	bdl	bdl	bdl	bdl	bdl	bdl
Se	57.8	bdl	bdl	bdl	bdl	bdl	bdl
Sn	5.8	bdl	bdl	bdl	bdl	bdl	bdl
Sr	1.7	6.98	bdl	7.74	bdl	10.63	bdl
Tl	0.6	bdl	bdl	bdl	bdl	bdl	bdl
U	0.6	bdl	bdl	bdl	bdl	bdl	bdl
V	5.8	bdl	bdl	bdl	bdl	bdl	bdl
W	1.7	bdl	bdl	bdl	bdl	bdl	bdl
Zn	5.8	bdl	21.03	3.94	14.00	57.91	bdl
Zr	11.6	bdl	bdl	bdl	bdl	bdl	bdl

bdl—below detection limit

## Results and discussion

### Origin and classification of airborne particles

The composition of PM in the Bochnia Salt Mine was analyzed at six carefully selected locations distributed along the atmospheric air flow. Morphological, mineralogical, and chemical characteristics were obtained using a combination of complementary analytical methods. Such a careful choice of sampling sites along the air flow path with a complete sampling and analysis approach was used for the first time. The results allowed to propose a genetic classification of the suspended matter present in the salt mine in the following three groups: geogenic, anthropogenic, and biogenic particles. The classification considers differences in morphology, phase, and chemical composition resulting from the origin of the particles. This genetic classification will allow in future for an optimal selection of monitoring methods, which will lead to a reduction in undesirable particles and an increase in the content of desired components (e.g., salt mist).

#### Geogenic particles

**Fig. 3:**
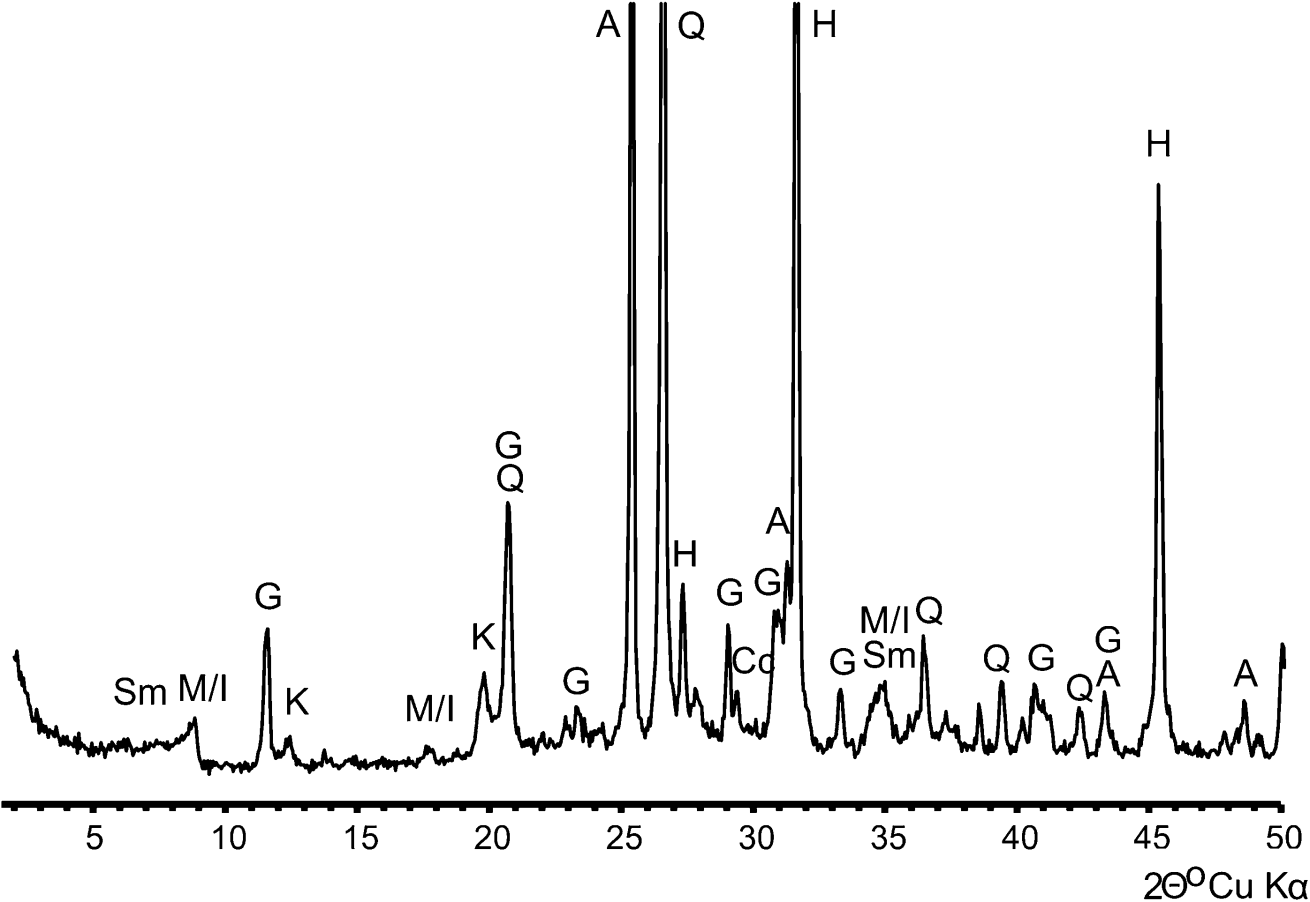
X-ray diffraction pattern of airborne particulate matter from the Bochnia Salt Mine. H—halite, Q—quartz, A—anhydrite, G—gypsum, M/I—muscovite/illite, Sm—smectite, K—kaolinite, Cc—calcite

Geogenic particles predominate the composition of airborne PM. They consist of natural fragments of minerals and rocks derived from the weathering and abrasion of surfaces exposed in the mine. Major minerals and mineral groups identified by XRD (Fig. 3) and characterized by SEM/EDS electron microscopy included halite, aluminosilicates, calcium sulfates (gypsum, anhydrite), quartz, and calcite.

**Fig. 4:**
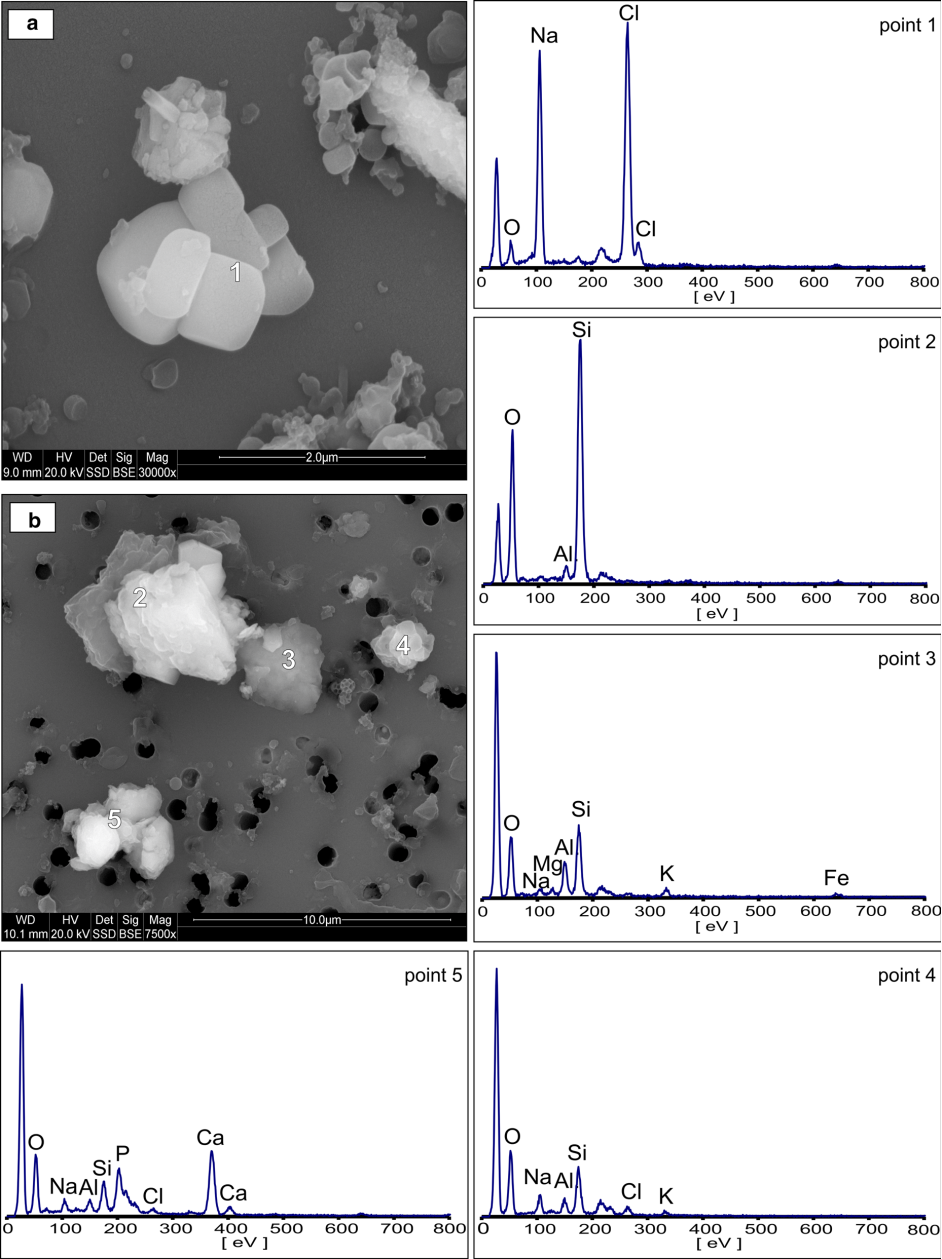
SEM images and EDS spectra of geogenic components: **a** halite (point 1), **b** quartz (point 2), aluminosilicates (points 3 and 4), and the aggregate of gangue rock (point 5). The unlabeled peak at low eV on the EDS spectrum is a polycarbonate filter artifact and this about 200 eV is due to the gold coating

**Fig. 5:**
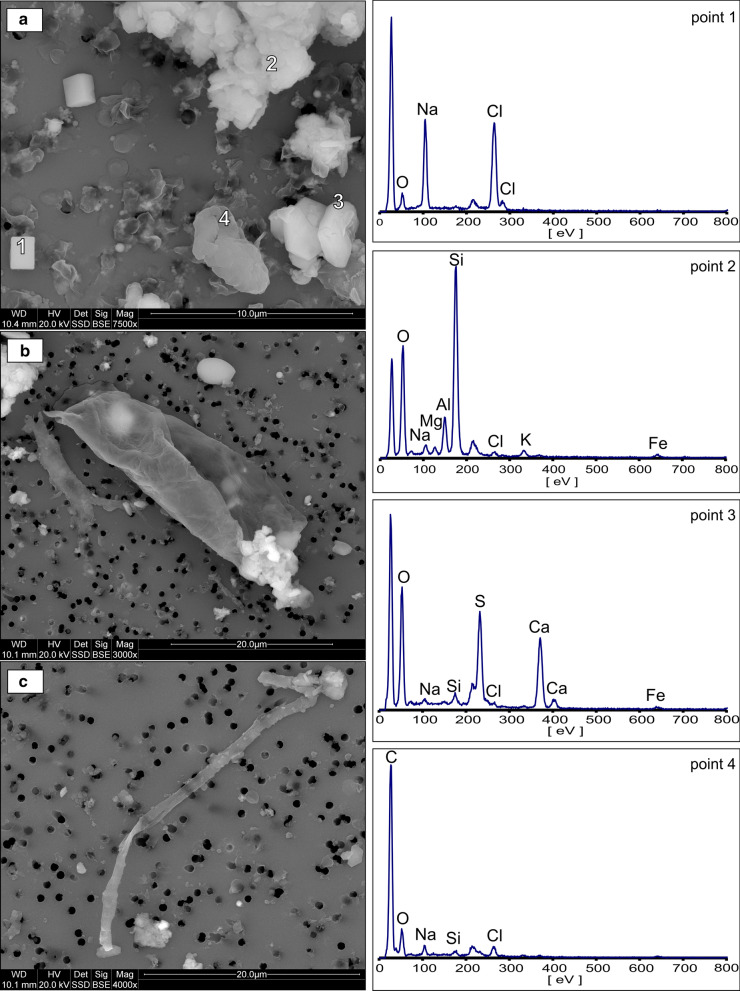
SEM images and EDS spectra of anthropogenic components related to the traffic of tourists: **a** rolled human skin flakes (point 4) associated with geogenic halite (point 1), mineral aggregate (point 2), and anhydrite (point 3); **b** flaky fragment of skin; c) organic textile fibers. The unlabeled peak at low eV on the EDS spectrum is a polycarbonate filter artifact and this about 200 eV is due to the gold coating

(i) Halite (NaCl). The presence of halite was demonstrated by XRD and SEM. The dominant morphology found for these particles was a characteristic regular cubic shape. This included not only fragments of rock salt but also halite precipitated from water aerosols. Particle size ranged from 1 to 4 µm. Figures 4a and 5a show micrographs of typical halite microcrystals with a smooth surface. Additionally, the presence of Na and Cl was detected in many mineral agglomerates (Figs. 4b and 5a). Halite particles were present in smaller amounts than calcium sulfates and aluminosilicates. This was most likely due to the high hygroscopicity of the halite. The RH was higher than 70%, which results in deliquescence of halite. This level of humidity was present at most measurement points. This aspect is discussed in more detail further in the section on chemical composition of PM. Rock salt components present in the form of a liquid aerosol escape the particulate analysis performed by air filtration. The methodology used here allows to detect only the less abundant solid form of NaCl in air. Therefore, the quantitative determination of the NaCl content in the air is underestimated here.
(ii) Aluminosilicates (Al–Si–O). XRD analysis indicated the presence of clay minerals such as smectites (for example, montmorillonite (Na,Ca)_0,3_(Al,Mg)_2_[(OH)_2_|Si_4_O_10_]·nH_2_O), micas and hydromicas (for example, illite (K,H_3_O)Al_2_[(OH)_2_|AlSi_3_O_10_]), kaolinite (Al_4_[(OH)_8_|Si_4_O_10_), and probably other layered aluminosilicates of similar structure and composition. The particles containing these constituents were usually in the form of aggregates with other mineral components of the deposit (Figs. 4 and 5). EDS analysis showed that the particles consisted mainly of Al and Si accompanied with Ca, Mg, K, and Fe. Varying amounts of Na and Cl were also present, indicating that the aggregates could be partly soaked with NaCl solutions from aerosol. Particles containing silicates and aluminosilicates were found in both TPM and PM_4_. This type of particles has a varied morphology, although they generally form irregularly shaped agglomerates. The size of agglomerates varied between 5 and 12 µm, while the size of individual particles ranged between 1 and 4 µm.
(iii) Ca-sulfates. Anhydrite (CaSO_4_) and gypsum (CaSO_4_·H_2_O), identified in the X-ray diffraction pattern, were common in both TPM and PM_4_ fractions. The SEM images showed characteristic crystalline particles containing only Ca, S, and O in the elemental composition apparent in the EDS spectrum (Fig. 5a). Particle size ranged from 1.4 to 5 µm. Such particles are also often found in slightly larger mineral aggregates.
(iv) Quartz (SiO_2_). Quartz is rarely observed in the form of distinct single particles. It usually occurs in aggregates with other mineral components. Quartz particles are 4–11 µm in size. Their presence in atmospheric dust was established by electron microscopy (Fig. 4b) and further confirmed by XRD. It is a natural component of the pelite in clay layers accompanying rock salt.
(v) Calcite (CaCO_3_). Small amounts of calcite were identified by XRD. It is an accessory component of the clay layers accompanying rock salt.

#### Anthropogenic particles

**Fig. 6:**
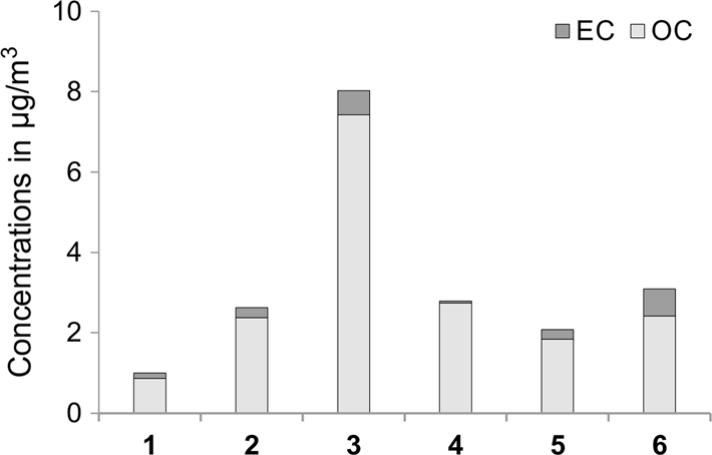
Concentration of organic carbon (OC), elemental carbon (EC), and total carbon (TC = OC + EC) at the consecutive sampling sites

This group includes mostly carbonaceous compounds that are associated with anthropogenic sources in outdoor and indoor environments (Goudarzi et al. 2018a, b, c; Amato et al. 2014). Previous reports relate indoor anthropogenic sources mainly to OC from organic textile fibers, cooking, and other organic emissions (Rivas et al. 2019). All these sources are possible here. The total carbon (TC) concentrations in TPM varied significantly between 1.01 µg/m^3^ in the air supply tunnel (site 1) and 8.08 µg/m^3^ in Ważyn Chamber (site 3) (Table 2). For comparison, TC concentrations of PM_10_ found in various indoor environments were higher and ranged from 20 to 50 µg/m^3^ (Fromme et al. 2008; Chithra and Nagendra 2013; Alves et al. 2014; Hu et al. 2015). SEM micrographs (Fig. 5) and carbon analysis (Fig. 6) showed that the relative increase in the levels of carbon at subsequent measurement sites was due to the emission of particles from tourist traffic and personnel activities in the mine (skin, hair, fragments of clothing, and pollutants deposited on their surface). A similar result was observed in the Wieliczka Salt Mine (Rogula-Kozłowska et al. 2016). However, the methodology used in this study allowed for more accurate identification of possible emission sources. For this group, four types of particulates were identified on the basis of different sources of emission.

##### Particles related to the traffic of people

The shedding of peeling human skin is considered to be an important contributor to indoor aerosols (Fox et al. 2008). This is an integral part of mass tourism. Indeed, the main sources of air pollution caused by the presence of humans identified by SEM in samples from several areas of the Bochnia Salt Mine were flakes of human skin (Fig. 5a, b). Particles from a several to several tens of µm were in the form of rolled flakes and fragmented fragments. Therefore, they both may be part of a finer PM_4_ fraction and contribute to an overall increase in TPM concentrations, in particular at sampling site 3. It should be noted that the number of visitors to the individual parts of the mine varies greatly and can fluctuate from day to day. Therefore, the once-off sampling on which the results of the TC, OC, and EC analyses are based (Table 2) should be considered as an approximation. The sampling campaign was conducted during the summer period in peak tourist traffic (at least 700 persons/day). It can therefore be assumed that these are the maximum concentrations of carbon in mine air from this source and are likely to be significantly lower at other times of the year.

The presence of skin flakes in airborne particles can contribute to the emission of bacteria belonging to the taxa that are closely related to the microbiome of human skin. The presence of bacteria was not measured at this stage of investigation. It was reported previously that in co-occupied rooms, people are exposed to bacteria from human skin (Qian et al. 2012; Amato et al. 2014). The results obtained by Frączek et al. (2013) in the Bochnia Salt Mine and by Myszkowska et al. (2019) and Gębarowska et al. (2018) in the Wieliczka Salt Mine confirmed that the isolated bacteria are typical commensal microorganisms that use the surface of the skin and respiratory mucous membranes as a natural reservoir and habitat. Nevertheless, because of the bacteriostatic effect of salt, the number of macro- and microorganisms in salt mines is significantly lower than that at the ground level (Gębarowska et al. 2018).

Mechanical ventilation in an underground mine can further contribute to the reduction of bacterial concentrations associated with human presence and thus reduce biological pollution. A decrease in the concentration of bacterial aerosols several hours after human presence was observed in the Bochnia Salt Mine by Frączek et al. (2013). Mechanically driven air movement can promote efficient air exchange and thus effective removal of pollutants (Wang et al. 2019). In addition, selected chambers can be ventilated with a separate fresh air stream, e.g., from level VIII, which can enhance the current circulation. This would involve drilling a vertical hole and blowing air up with a fan.

The ability to control the air exchange ratio in an underground salt mine can be a significant advantage compared to the artificial halochambers on the surface. For artificial halochambers, more care is taken to produce the appropriate saline aerosol than to ventilate this closed inner space properly (Chervinskaya 2007; Sandu et al. 2013, 2018).

##### Soot particles

**Fig. 7:**
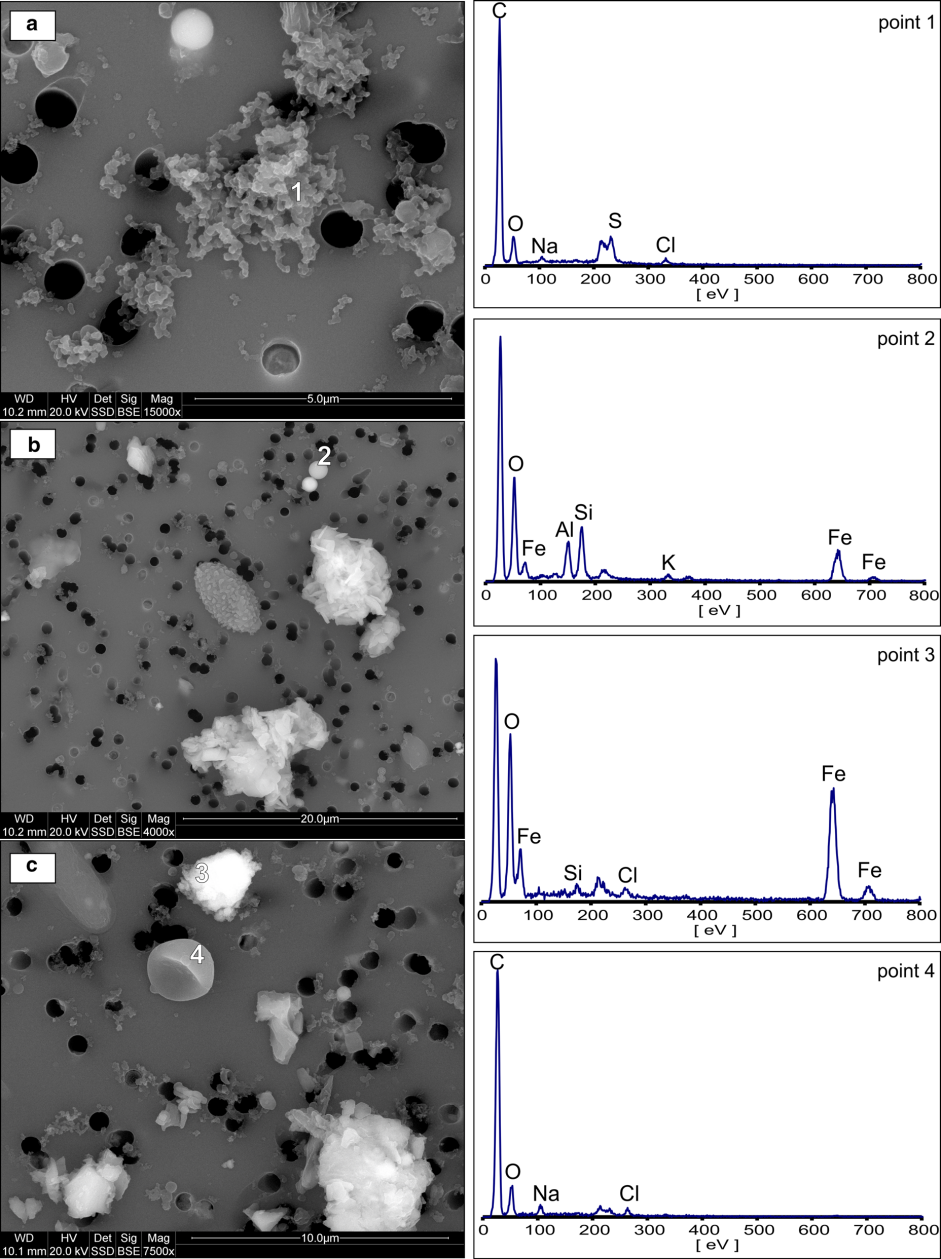
SEM images and EDS spectra of anthropogenic components (from industrial and local sources) and biogenic components. **a** Soot aggregates (point 1), **b** industrial particles (fly ash, point 2), **c** iron oxide (rust, point 3) and pollen (point 4). The unlabeled peak at low eV on the EDS spectrum is a polycarbonate filter artifact and this about 200 eV is due to the gold coating

Soot particles, also known as black carbon, occur in small quantities. The presence of these particles at sampling point 1 suggests their intake with air flow from outside through a ventilation shaft. Soot particles form elongated aggregates of very fine spherical particles of 15–50 nm in diameter (Fig. 7a). Black carbon is often considered as a product of combustion of fossil fuels and biomass (Cong et al. 2009). However, considering the summer season during sampling as well as the size of the particles and the morphology of soot aggregates, they are most likely emitted from vehicle exhaust. Similar opinions on traffic-related soot particles have also been expressed elsewhere (Liati et al. 2016; Michalik et al. 2016; Faimon et al. 2019; Islam et al. 2019).

The EC content can be considered as an indicator of air pollution entering the mine through the ventilation system. An increase in EC concentration was, however, observed at subsequent measurement points in relation to the values of the incoming air (sampling point 1). This indicates the existence of internal sources of EC in the mine. Most probably, some elemental carbon is brought in by tourist traffic. The process of introduction of municipal aerosol (including fine black carbon) by visitors on clothing, hair, and skin was reported for the show cave (Jeong et al. 2003; Chang et al. 2008).

The EC concentration ranged between 0.13 µg/m^3^ in the air supply tunnel and 0.68 µg/m^3^ in the furthest sampling point (Table 2). The average EC concentration in the Bochnia Salt Mine was 0.32 µg/m^3^, which is comparable to the background level determined during summer season in rural areas in Poland (Rogula-Kozłowska et al. 2014; Klejnowski et al. 2017). The concentration of EC in PM_2.5_ particles sampled during summer season in urban areas of Poland is several times higher (Rogula-Kozłowska et al. 2012, 2014). Hence, apart from the presence of soot particles, the concentration of the EC fraction in the Bochnia Salt Mine is extremely low. However, a higher EC content in PM of a salt mine cannot be ruled out, particularly in winter, as significant seasonal changes in the carbon content in the ambient particulate matter are observed in Poland (Sówka et al. 2019; Samek et al. 2020). The results obtained in this study indicate that the distance from the ventilation shaft is one of the main factors in the air purification process of the ambient air pollutants sucked into the mine from the surface. Therefore, in the case of elevated concentration of carbon in polluted atmospheric air on the surface, the areas available to the public in the Bochnia Salt Mine are in a privileged position due to a very long distance (over 1000 m) from the ventilation shaft to the entrance of the tourist area. This allows the air to be cleaned of particles containing EC.

##### Industrial particles

Industrial particles are mainly represented by fly ash. It is manifested by the presence of spherical particles consisting of Si and Al, often with a small admixture of Fe (Fig. 7b). Sporadically, there are also spherules composed exclusively of Fe. The size of fly ash particles ranges from less than 1 to 1.5 µm. These particles are sucked down with the air from the surface. This type of air pollution is transported by winds as long range transport from large agglomerations and industrial centers located nearby in Kraków, Tarnów, and Upper Silesia (Wilczyńska-Michalik et al. 2014; Jabłońska and Janeczek 2019). Similar to soot particles, fine or very fine aluminosilicate spheres were found mostly near the ventilation shaft and disappeared with the distance. Because of the air purification process, the air pollutants present on the ground in the form of fly ash are observed in a salt mine in very limited quantities and do not pose a threat to the human respiratory system.

#### Metallic particles from local sources

Occasionally, airborne Fe-rich particles and aggregates were observed (points 2 and 5). An example of an iron oxide particle is shown in Fig. 7c. No potentially toxic metals were detected in PM by the SEM/EDS method. Iron oxides are probably the rust chips from railway tracks, steel arch supports, tools, and equipment. Appropriate frequent cleaning and ventilation reduce the concentration of these particles associated with metal corrosion.

#### Biogenic aerosol

In the Bochnia Salt Mine, pollen grains were occasionally observed, and their number and size significantly decreased with the distance from the ventilation shaft. This is an additional evidence of the progression of the self-cleaning process through the air flow. The content of pollen and fungal spores, regardless of the sampling site, was very small and did not pose any risk to allergic persons. During the sampling, only sporadic fungal spores and pollen grains were recorded (Fig. 7). These results are consistent with the results of tests conducted by different methods at the Wieliczka Salt Mine (Myszkowska et al. 2019). The presence of pollen grains and fungal spores mainly in samples collected in the air supply tunnel suggests that they originated from outside. This contradicts some views that pollen grains are brought unintentionally by humans into the areas of the mine exclusively used for speleotherapy (Cristofori et al. 2020; Myszkowska et al. 2019).

### Distribution of the concentration of airborne particles

**Fig. 8:**
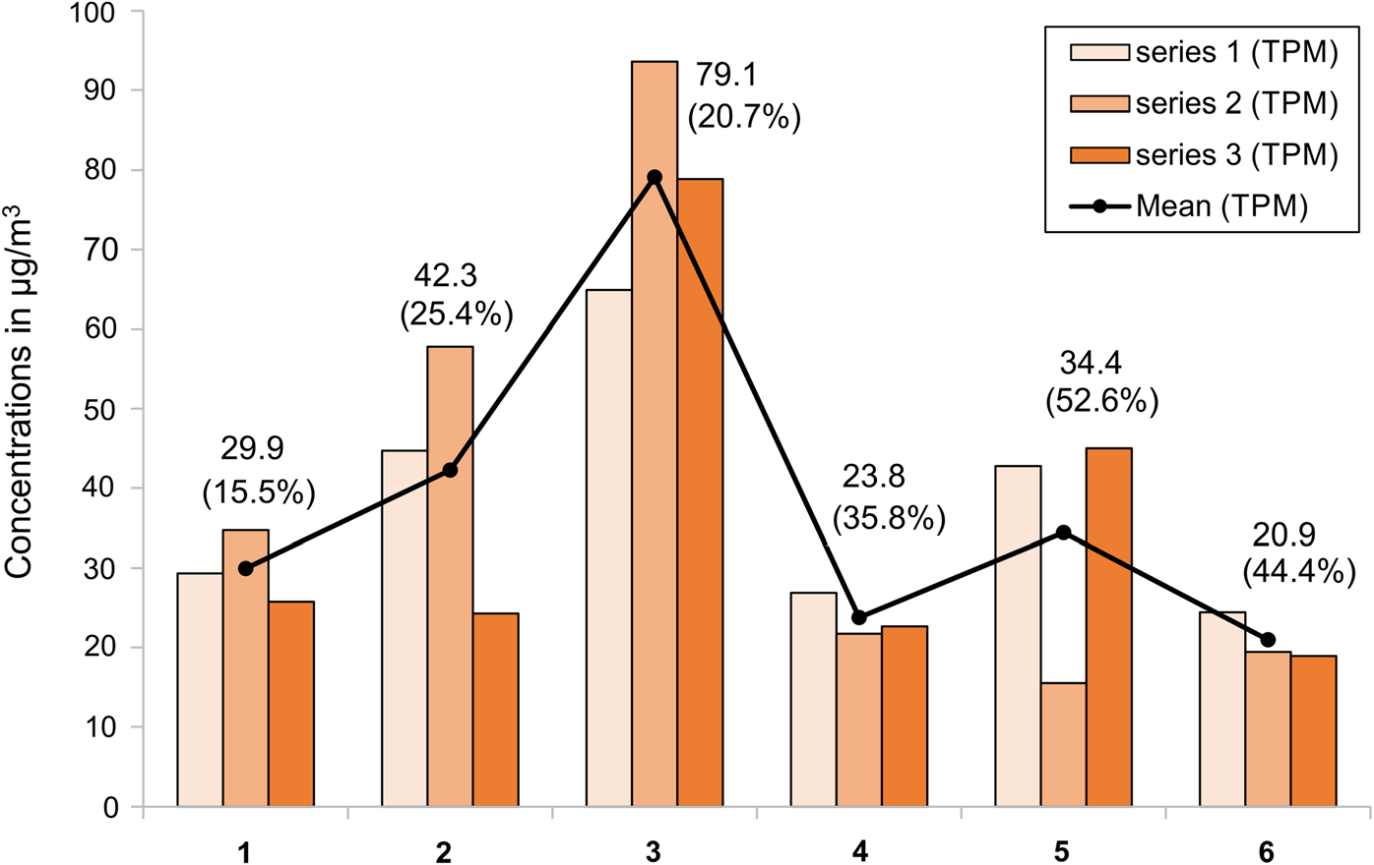
Distribution of total particulate matter (TPM) concentrations in the air of the Bochnia Salt Mine at consecutive measurement sites determined by three sampling campaigns. The numbers indicate the mean value of TPM and the percent contribution of PM_4_

Figure 8 shows the results of three consecutive measurements of total suspended dust concentration (TPM) conducted at six different locations in the mine. All the measurements were completed in June 2019 using the same method; thus, the possible impact of seasonal variations is minimized. The points on the diagram are arranged in the order of increasing distance from the air intake to the mine. At point 5, two air streams with different transport distances merge. Points 2 to 6 are located on tourist trails. The highest tourist traffic occurs at points 2 and 3.

The variations between the results of the three consecutive measurements partly reflect the daily variations that result from varying intensity of tourism and activities. The differences between the three measurements repeated at point 6 best reflect the repeatability of the method. This point, farthest from the air intake and the main tourist route, has the most stable atmospheric composition. The standard deviation determined from the three readings at this point did not exceed 15% and was significantly smaller than fluctuations at other locations and variations between the sampling points. The fluctuations between the three measurements observed at points 2, 3, and 5 best reflect the day-to-day variation resulting from varying intensity of tourist traffic and maintenance works carried out in the mine.

The highest concentrations of TPM were recorded at points 2 and 3 (average values of 42 and 79 μg/m^3^ air, respectively). This is due to the tourist traffic and the activities carried out in the mine, as these are the two busiest locations. Local increase in dust concentration related to the number of visitors is observed in many tourist facilities, namely museums (Worobiec et al. 2008; Krupińska et al. 2012), caves (Smith et al. 2012; Grgić et al. 2014Licbinsky et al. 2020), and salt mine (Salmon et al. 1996). Tourist services also require transport (underground railway) and work related to safety and maintenance (repairs, maintenance, daily cleaning, etc.). These works cause local and short-term re-suspension of dust. Cleaning and sweeping commonly cause an increase in dustiness (Corsi et al. 2008; Lewis et al. 2018). In the Ważyn chamber (sampling point 3), apart from daily tourist traffic, overnight stays and many special events are organized. Consequently, this location is particularly exposed to periodic increase in TPM concentrations. Furthermore, a tourist train running from the Campi shaft to the Sutoris shaft can increase the amount of airborne particles measured at sampling points 2 and 5. At subsequent measurement locations, the TPM concentration decreased with the distance from the intake well, reaching average values comparable to those measured in the supply tunnel. This indicates gravitational deposition of PM within the mine.

The concentration of PM_4_ was particularly low at the first three points (not exceeding 26% TPM). The further three locations showed slightly higher values (36%, 53%, and 44% TPM). This is likely to be partly due to the mine’s operations and partly due to an increase in the proportion of mineral salts and aerosols.

### Distribution of chemical composition of airborne particles

#### Water-soluble ions

**Fig. 9:**
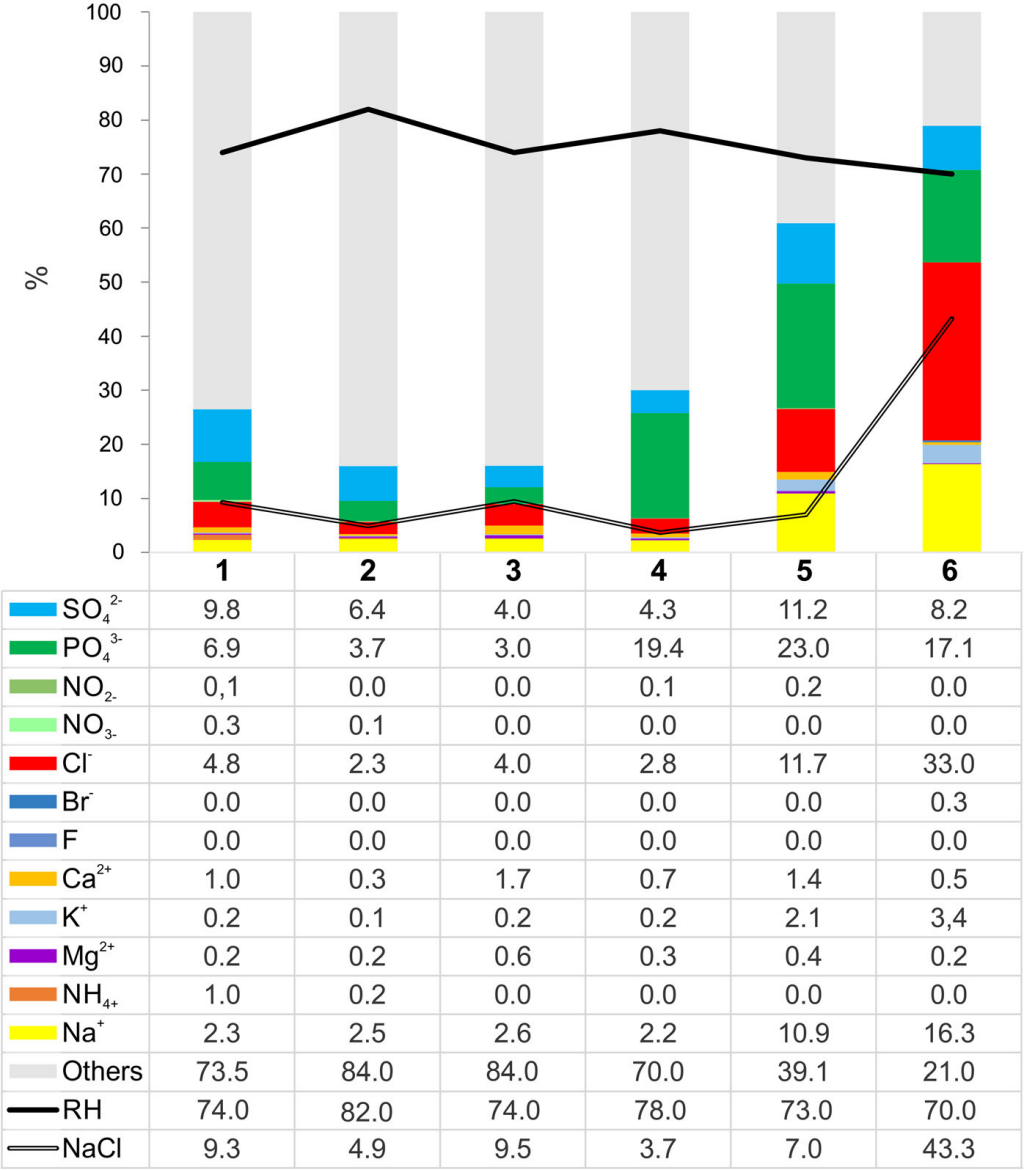
Mass percentage of water-soluble ions in TPM sampled at the consecutive sites. The figure also shows relative humidity (RH) and mass percentage of NaCl in TPM calculated using the stoichiometric method based on the concentration of Cl ions. Others are carbonaceous fraction, microelements and undetermined water-insoluble components

Ion concentrations are shown in Table 2. The results of the analyses showed that the main water-soluble ions in all PM samples were chlorides, sodium, sulfates, and phosphates. The content of calcium, magnesium, potassium, and ammonium ions was much smaller. The soluble fraction represented 16% (site 3) to 79% (site 6) of the total mass of PM (Fig. 9).

The presence of Na and Cl derived from salt rocks in underground salt mines as well as from marine aerosol in coastal locations is a key factor for the therapy of ailments of respiratory airways. This is in contrast to inland spray, where Na^+^ and Cl^−^ fractions are mainly related to biomass combustion and road traffic (Samek et al. 2018; Sówka et al. 2020). Because of the hygroscopic behavior of NaCl, the concentration of solid NaCl aerosol strongly depends on the atmospheric RH (Tang and Munkelwitz 1993). Only at a very low values of RH, atmospheric aerosol particles containing inorganic salts are solid (Seinfeld and Pandis 2006). This has a significant effect on the measurement of the concentration of inorganic salts in the salt mine aerosol sampled by filtration. The RH at which solid particles spontaneously absorb water and form a saturated solution droplet by water vapor condensation is known as deliquescence RH (DRH). NaCl aerosols have a deliquescence point at 75 ± 2% RH (Tang and Munkelwitz 1993). In addition, the DRH of multicomponent salt aerosols (which are expected in salt mines) is lower than that of the individual salts (Salmon et al. 1996; Seinfeld and Pandis 2006). The liquid dripping from surfaces of the mine corridors was observed during material sampling for this research. Thus, aerosol components originating from rock salt are partly present in liquid form. The dependence of the measured ion concentrations on RH is shown in Fig. 9. Decreasing RH values correlated with increasing NaCl concentrations. RH values measured in the Bochnia Salt Mine ranged from 70 to 82% and temperature ranged from 14 to 20 °C. Even at lower values of RH (< 75%), a single salt can occur in one of the two states: as a solid or as an aqueous solution (Seinfeld and Pandis 2006). The effect of salt deliquescence in humid air can be observed on numerous occasions on the ceilings and walls of salt mine excavations.

Therefore, the concentration of ions estimated in this work by using only filter-based methodology is underestimated. Under the conditions of a salt mine with a high RH regime, a more accurate insight into the concentration of inorganic salts in the air aerosol can be gained from other methods, e.g., the “scrubbing” method used by Kostrzon et al. (2017) at the Wieliczka Salt Mine. They showed that the concentration of NaCl measured by them (3700 µg/m^3^) was several times higher than that determined at the same location by Rogula-Kozłowska et al. (2016) by the filtration method (approximately 14 µg/m^3^).

#### Microelements

**Fig. 10:**
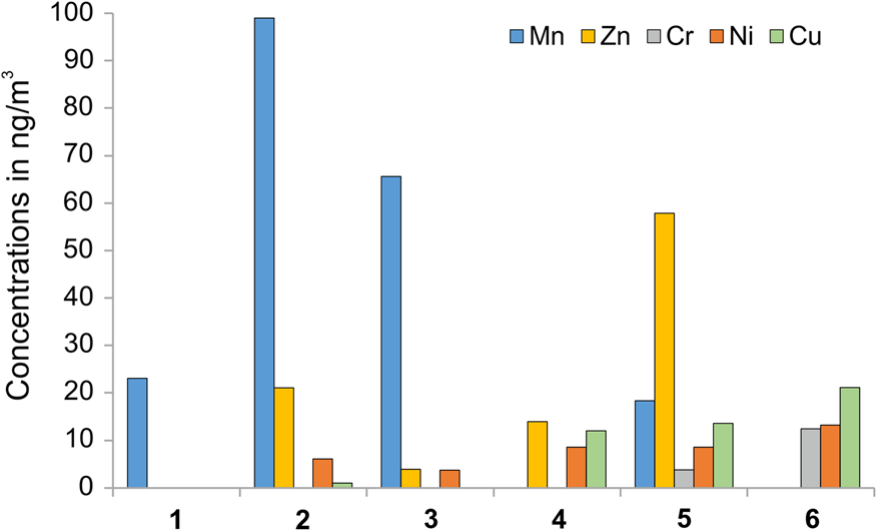
Selected elemental concentrations sampled at the consecutive sites

The dominant elements in PM are Fe, Al, Ag, Mn, and Zn. Fe and Mn were found to be at the highest concentration at the second sampling point (10,209 ng/m^3^ and 99 ng/m^3^, respectively) and the third sampling point (5389 ng/m^3^ and 66 ng/m^3^, respectively) (Table 2). The measured concentrations of Fe at all sites except sites 4 and 6 and the measured concentrations of Mn at sites 2 and 3 can be considered as high. At the subsequent sampling points, the concentration of these components gradually decreases probably due to gravitational deposition (Fig. 10 and Table 2). The highest level of Zn was recorded at the fifth sampling point (58 ng/m^3^). The ranges of Mn and Zn concentrations determined in the remaining samples at the Bochnia Salt Mine were lower than those determined in the air dust collected on the surface at several locations in Poland (Rogula-Kozłowska 2012; Zwoździak et al. 2013; Samek et al. 2020) and were similar or lower than those in the samples collected from indoor spaces (Worobiec et al. 2008; Mainka et al. 2015).

The presence of potentially toxic elements such as Pb, As, Cd, Hg, and others metals listed in Table 2 was not detected. However, the analysis revealed the presence of trace amounts of Ni, Cu, and Cr. As shown in Fig. 10, their content gradually increased along the atmospheric air flow in the mine, reaching up to 12 ng/m^3^ for Ni and Cr and 21 ng/m^3^ for Cu at the farthest sampling site. Nevertheless, these concentrations of Ni, Cr, and Cu were below than European Union air quality standards (EU 2004) or below the indoor concentrations observed in Poland (Worobiec et al. 2010; Mainka et al. 2015).

Although traces of Fe, Mn, Zn, Cu, and Co are considered essential for all living organisms (Steinnes, 2009; Geiger and Cooper, 2010), elevated levels of metals are generally associated with a wide range of negative environmental and health effects, including respiratory and lung disorders (Goudarzi et al. 2018a, b, c; Ali et al. 2019; Dastoorpoor et al. 2020). In air, metals are mostly associated with the presence of solid particles of anthropogenic origin (Idani et al. 2018). A human health risk assessment established by the US Environmental Protection Agency (US EPA 2009) is a widely used approach to estimate the nature and probability of adverse health effects on humans who may be exposed to chemicals. It is therefore important to continuously monitor not only the concentration of water-soluble ions but also the metals carried by PM in the air in salt mines open to the public, especially in underground excavations intended for sanatoriums.

#### Source identification

PM consists of a mixture of mineral grains in varying proportions, complex mineral agglomerates, single mineral grains, coatings on other particles, etc. The chemical composition of the sample clearly depicts a specific group of minerals and some local sources of metal emissions, which was verified on the basis of analysis of individual particles by XRD and SEM. Elevated concentrations of Mg^2+^, Ca^2+^, Al, and Ti indicate an increasing proportion of clays. Appropriate increase in Na and Cl ions content indicates higher content of halite from the deposit (Table 2). For the above-mentioned components of geogenic origin, high variability of concentrations was found (Table 2). This is most probably due to the dimensional pattern of the layers in the parent rocks exposed in the mining walls. The occurrence of sporadically increased concentrations of some elements may suggest that they come from resuspended rock dust. This is clearly visible in the Ważyn Chamber (site 3), where increased concentrations of some components correlated with increased TPM. Therefore, the distribution of these elements in subsequent locations is related to local sources originating from activities leading to the re-suspension of dust, i.e., clean-up activities or physical activity of visitors. Moreover, the mineral composition of the settled dust may vary depending on the geological conditions in the mine.

An increasing proportion of minerals from the deposit, although not so obvious, was observed for Ca^2+^, SO_4_^2−^, and Sr. These components showed the presence of anhydrite or gypsum in TPM, but the interpretation of their appropriate concentration is much more difficult, as Ca^2+^ and SO_4_^2−^ are also constituents of other components in the air dust. Under other conditions (e.g., on the surface), the presence of SO_4_^2−^ ions together with NH_4+_ and NO_3_^−^ can be interpreted as air pollution (Styszko et al. 2017). However, our research shows that components of the pollutants common to the outside air, including NH_4+_ and NO_3_^−^, are rapidly removed from the air in the Bochnia Salt Mine (Table 2). In salt mines where sulfate minerals are naturally present, SO_4_^2−^ ions are naturally derived from weathering and abrasion of the parent rock containing sulfate minerals. Because anhydrite present in the Bochnia deposit contains Sr and because gypsum crystals are often accompanied by a small amount of celestine SrSO_4_, the correlated increase in the mass proportion of SO_4_^2−^ and Sr seems to be an unambiguous determinant of the presence of gypsum and anhydrite particles in PM. This is consistent with SEM results.

An increase in the proportion of phosphates in TPM was observed in the Ważyn Chamber, where the underground kitchen is located. This is probably due to the small amount of smoke from frying food as well as fumes from cleaning agents. As mentioned earlier for pollution associated with tourist traffic, an appropriate ventilation system limits this phenomenon.

Trace metals detected in PM (Table 2) are also present in nature and are released into air from both natural sources and human activities. However, certain types of metals and their elevated concentrations in underground salt mine are a result of human activity and maintenance work. The concentrations of Fe, Mn, and Zn (partly) were correlated. Fe and Mn were found to be at the highest concentration at the second sampling point where the underground railways are located, indicating track corrosion as the main emission source. An increase in Zn concentration at the fifth sampling point was due to the presence of galvanized steel pipes used for air flow control and compressed air transport in this area. In addition, the increasing trend of concentration of some trace metals such as Ni, Cu, and Cr suggests their origin related to the construction material used in the older eastern part of the mine, e.g., pipes, cables, and transformers. Metal corrosion in such an environment of a salt mine is accelerated compared to that in other places and unfortunately cannot be avoided. Thus, if it is possible, special care should be taken in selecting the construction material in salt mines, for example using only timber supports, and all damaged and corroded cables and equipment should be replaced by new ones, while the old metal pipes should be replaced by PVC pipes. Fortunately, the content of trace metals in PM was small and did not pose any risk to human health.

## Conclusions

A detailed genetic classification of airborne particles present in an underground salt mine was proposed and comprised three main groups: geogenic, anthropogenic, and biogenic particles. Geogenic particles predominate and include fragments of rock salt and associated minerals. The anthropogenic group includes mostly carbon-containing particles that are typical for indoor air in the areas with tourists traffic and pose no risk to health. Biogenic component, represented by pollen, decreases with the distance from the ventilation shaft as an evidence of the progression of the self-cleaning process through the air flow. This classification will allow for an optimal selection of monitoring methods. More importantly, it will help to actively reduce the content of potentially undesirable particles and increase the content of desired components in (e.g., salt spray) in future.

For the first time, a comprehensive study of the distribution of airborne particulates along the flow of atmospheric air in an underground salt mine was conducted. The research was performed in an underground salt mine, which, similar to other decommissioned salt mines in the world, currently focuses on tourism, recreation, and health. Quantitative morphological, mineralogical, and chemical characteristics of particulates were obtained and compared. The total concentration of PM, undisturbed by the activities conducted in the mine, can be considered to be low. The air in the salt mine does not contain particles and chemical components at concentrations that would be toxic to humans or have a negative impact on the health of visitors. The primary component of the aerosol is natural material derived from the weathering of the parent rock. These geogenic particles (halite, aluminosilicates, anhydrite, gypsum, quartz, and calcite) are dominant. Neither the mineral composition and particle morphology nor the amount of this material is hazardous to human health. In contrast, the formation of native aerosol in a mine creates a unique environment that is difficult to achieve in surficial environments where anthropogenic particles predominate.

A local increase in dust concentration and emission of typical indoor particles (e.g., fragments of skin, hair, and clothing) is observed in the areas of increased tourist traffic and activities in the mine. This is typical for indoor spaces and does not exceed levels observed elsewhere. In addition, the process of dry deposition of particulate matter inside the mine and simultaneous appropriate ventilation system can effectively ensure adequate air purity in underground chambers. The self-purification process can be enhanced during periods of reduced human activity (e.g., at night) or through technical operations (e.g., appropriate cleaning procedures and a rigorous ventilation system).

Although some of the particles common to outdoor air, such as soot, biogenic materials, and industrial particles, are drawn into the mine from outside (and partly brought by tourists), their content is so small that they pose no risk to humans. It was observed that the amount of these particles decreases with the distance from the ventilation shaft (air intake to the mine). A higher content of anthropogenic particles associated with the combustion of fossil fuels in winter cannot be excluded. Therefore, future studies should examine the effect of seasonal differences. However, a very long distance from the ventilation shaft to the entrance of the tourist area in underground salt mines will probably allow sufficient purification of potentially polluted atmospheric air from the surface. Therefore, one of the most important factors in the process of selecting underground chambers for sanatorium purposes should be the distance from the ventilation shaft. It is also very desirable to ventilate the chambers with an additional stream of air, which may be conducive to the effective removal of pollutants typical for indoor spaces.

Because underground salt mines are commonly used for halotherapy, the composition of air aerosols was also assessed from a human health perspective. Similar to previous reports from the other salt mine (Rogula-Kozłowska et al. 2016; Kostrzon et al. 2017), mineralogical analysis showed that the air aerosol in the Bochnia Salt Mine contains particles of halite. Various forms of NaCl also accompany other air components, which was confirmed by chemical analyses. However, the quantity was underestimated in this study due to the application of filter-based methodology. The presence of salt, which may have anti-inflammatory and antiallergic properties, is beneficial to human health (Lazarescu et al. 2014; Kalaci et al. 2019). Such an aerosol is considered as a therapeutic factor in halotherapy. The presence of salt spray in the atmosphere indicates the possibility of further development of sanatorium activities in underground salt mines.

The air in the underground corridors of salt mines, in addition to halite and other salt aerosols, may also contain several amounts of other evaporate minerals (e.g., anhydrite, gypsum) and fragments of terigenic minerals (e.g., clays, quartz) which in small quantities pose no risk to human health. In addition, the operation and corrosion of machinery and construction materials used in the mine may introduce small quantities of undesirable components (rust, metal compounds) into the atmosphere. The potential positive effects or risks associated with these substances should be considered together with the simultaneous presence of various types of particles. The proposed detailed genetic classification of airborne particles present in underground salt mines is aimed to facilitate this task. The challenge for all mining facilities dedicated to halotherapy is the need to adapt them to serve tourists while maintaining air purity and preserving the presence of native, desirable salt aerosols. The results of our research support the need to regulate the legal requirements for air quality standards in facilities used for halotherapy in all countries where these treatments are traditionally recognized in medicine. Sanatoriums and halotherapy units arranged in properly prepared halls and chambers in salt mines will also enable high-quality research to be conducted in the future to provide more detailed data on the effectiveness of this therapy.
